# Protective and Susceptibility Effects of Human Leukocyte Antigen on Melanoma Prevalence and their Implications for Predicting Checkpoint Blockade Immunotherapy Outcomes

**DOI:** 10.29245/2578-3009/2022/2.1238

**Published:** 2022-06-29

**Authors:** Lisa M. James, Apostolos P. Georgopoulos

**Affiliations:** 1 The HLA Research Group, Brain Sciences Center, Department of Veterans Affairs Health Care System, Minneapolis, MN, 55417, USA; 2 Department of Neuroscience, University of Minnesota Medical School, Minneapolis, MN 55455, USA; 3 Department of Psychiatry, University of Minnesota Medical School, Minneapolis, MN 55455, USA; 4 Department of Neurology, University of Minnesota Medical School, Minneapolis, MN 55455, USA

**Keywords:** Melanoma, HLA, Cancer epidemiology, Immune blockade immunotherapy

## Abstract

The association of Human Leukocyte Antigen (HLA) with melanoma has been well documented. Similarly, the outcome of checkpoint blockade immunotherapy (CBI) in melanoma depends, to some extent, on the HLA genotype of the patient. Although specific favorable (or unfavorable) HLA alleles for CBI outcome for melanoma have been identified, there is currently no reliable way to predict a positive, neutral or negative melanoma CBI outcome for other alleles. Here we used an immunogenetic epidemiological approach to identify HLA alleles whose frequency is negatively (or positively) associated with melanoma prevalence (protective or susceptibility alleles, respectively). The findings demonstrated that, indeed, HLA alleles that are negatively associated with melanoma prevalence in the population have been associated with good CBI outcome at the individual level and, conversely, HLA alleles that are positively associated with melanoma prevalence have been associated with poor CBI outcome in individuals. Given this good prediction of CBI cancer immunotherapy by specific immunogenetically discovered HLA alleles, we used this epidemiologic immunogenetic approach to identify more HLA Class I and II alleles protective (or susceptibility) for melanoma which would thus be good predictors of CBI outcomes in those cancers. This is a new approach to successfully (a) identify HLA protective or susceptibility alleles for melanoma, and (b) use that information in anticipating outcomes in CBI cancer immunotherapy.

## Introduction

The association of human leukocyte antigen (HLA) with cancer in general^1,2^ and melanoma in particular^3^ has been well documented. Research has been mainly focused on the role of HLA class I and associated engagement of CD8+ cytotoxic T lymphocytes in eliminating tumor cells under the hypothesis that novel antigens produced by tumor cells (“neoantigens”)^4,5^ attach to HLA class I molecules, forming a complex that moves to the cell surface, where it is recognized by CD8+ T lymphocytes resulting in cell death and apoptosis. This mechanism is thought to be suppressed by substances secreted by tumor cells which suppress T cell activation^6,7^. In fact, CBI is thought to act by blocking this tumor-induced T lymphocyte suppression, thus allowing CD8+ T lymphocytes to recognize HLA class I molecule – neoantigen complexes and kill the tumor cell. Under those conditions, the therapeutic effectiveness of CBI would depend on how well the HLA-neoantigen complex can engage the CD8+ lymphocyte in the first place. In that context, it was found^8^ that CBI produced a good outcome in melanoma patients with certain HLA class I alleles (B*18:01, B*44:02, B*44:03, B*44:05, B*50:01), whereas it had a poor outcome in patients with the B*15:01 allele. This differential therapeutic effect was attributed to how well those alleles may bind to melanoma tumor neoantigens^8^. It is reasonable to suppose that HLA binding affinity to melanoma tumor neoantigens would have consequences for the general population, outside of melanoma CBI therapy. More specifically, we hypothesized that such a mechanism could operate at the population level with the consequence that alleles that have been shown to have a positive effect on CBI would be associated with lower melanoma prevalence (protective alleles), whereas alleles that have been shown to have a negative effect on CBI would be associated with higher melanoma prevalence (susceptibility alleles). We tested this hypothesis by evaluating the correspondence between the population frequency of HLA alleles that have been shown to influence the outcome of CBI prevalence and the population prevalence of melanoma in 14 Continental Western European (CWE) countries. In addition, we extended those analyses to evaluate the association of additional HLA class I and class II allele frequencies in CWE (127 alleles in total) with the population prevalence of melanoma to identify HLA alleles that, at the population level, are associated with susceptibility to or protection against melanoma.

With respect to HLA class II alleles, they have also been involved in cancer in general^9^ and melanoma in particular^3^. Two main explanations have been advanced regarding the role of HLA class II in cancer. One is direct, involving the production of antibodies against tumor neoantigens and subsequent elimination of tumor cells; the other is indirect, based on the finding that CD4+ T lymphocytes activated by the HLA class II molecule – neoantigen complex induce proliferation of CD8+ T lymphocytes through the release of IL-2^6,7,9^. In addition, HLA alleles may influence cancer via elimination of cancer-inducing viruses and bacteria^10,11^. Both the production of antibodies against tumor neoantigens and the elimination of cancer-inducing pathogens rests on the ability of HLA alleles to first bind with antigens. Subtle alterations in the HLA binding groove alter binding affinity^12,13,^ thereby influencing the scope of antigens that can be bound and eliminated. Thus, characterization of HLA alleles as protective or susceptible with regard to the population prevalence of melanoma is presumed to reflect, in part, binding, and therefore elimination of, antigens that could otherwise contribute to melanoma.

## Materials and Methods

### Melanoma prevalence

The population prevalence of melanoma was calculated for 14 CWE countries including Austria, Belgium, Denmark, Finland, France, Germany, Greece, Italy, Netherlands, Norway, Portugal, Spain, Sweden, and Switzerland. Specifically, the 2016 melanoma case counts as determined by the Global Burden of Disease study^14^ were divided by the 2016 total population for each country^15^.

### HLA

The population frequencies of class I (A, B, C) and class II (DRB1, DQB1, DPB1) classical genes in each of the 14 CWE countries were retrieved from Allele Frequency Net Database^16,17^, a public repository of immune gene frequencies worldwide. Of the 844 distinct alleles, 127 occurred in 9 or more of the 14 CWE countries above and were used in subsequent analyses. The distribution of the alleles used to Class and Gene is given in Table 1.

### HLA Class I supertypes

Alleles of Class I A and B genes were assigned to a supertype^18^. (Supertypes for gene C of Class I or any gene of Class II have not been described.) Of a total of 56 alleles of Class I A and B genes, 53 alleles could be assigned to supertypes based on the assignments provided by Sidney et al.^18^, namely all 20 A gene alleles and 33/36 B gene alleles; B*13:02, B*47:01 and B*49:01 were unassigned (Fig. 2 in Sidney et al.^18^). The distribution of the 56 HLA Class I alleles to supertypes is given in Table 2. The individual alleles used and their assignments to Class, Gene and Supertype are given in Table 3.

### Data analysis

The primary analysis was the calculation of the raw melanoma score, namely the Pearson correlation coefficient r between the population prevalence of melanoma and the population frequency of each of the 127 HLA alleles. Fisher’s z-transformation^19^ was applied to r normalize its distribution:

Immunogenetic melanoma score (IMS)

(1) $$r^{\prime }=\mathrm{atanh}(r)\;\;=\frac{1}{2}\text{ln}\left(\frac{1+r}{1-r}\right)$$

We have used this measure in previous studies of immunogenetic epidemiology of neurodegenerative diseases^20-25^, type 1 diabetes^26^, and various types of cancers^27,28^. The effects of HLA class and genes on the proportion of protective alleles in the population were evaluated using the Wald test for a single proportion (2-sided) and by constructing Agresti-Coull 95% confidence intervals (CI) for the proportions.

Since each individual carries 2 alleles for each one of the 6 genes, for a total of 12 alleles, we also derived an expected estimate, R’, of melanoma protection/susceptibility for each allele as follows. For a given allele, we retained its r’ and obtained the remaining eleven r’ allele values by randomly drawing with replacement from the pools of alleles of each gene. This process was repeated 1000 times, and an average R’ was calculated from 12x1000=12000r’ values (i.e. the value for the specific allele under consideration and the 11 remaining randomly drawn values, x1000). Finally, R’ was computed for each one of the 127 alleles. Analyses were conducted using SPSS (Version 27) and Intel Fortran (version 16.8.3).

## Results

### Melanoma immunogenetic scores: Individual alleles

**Figure 1:**
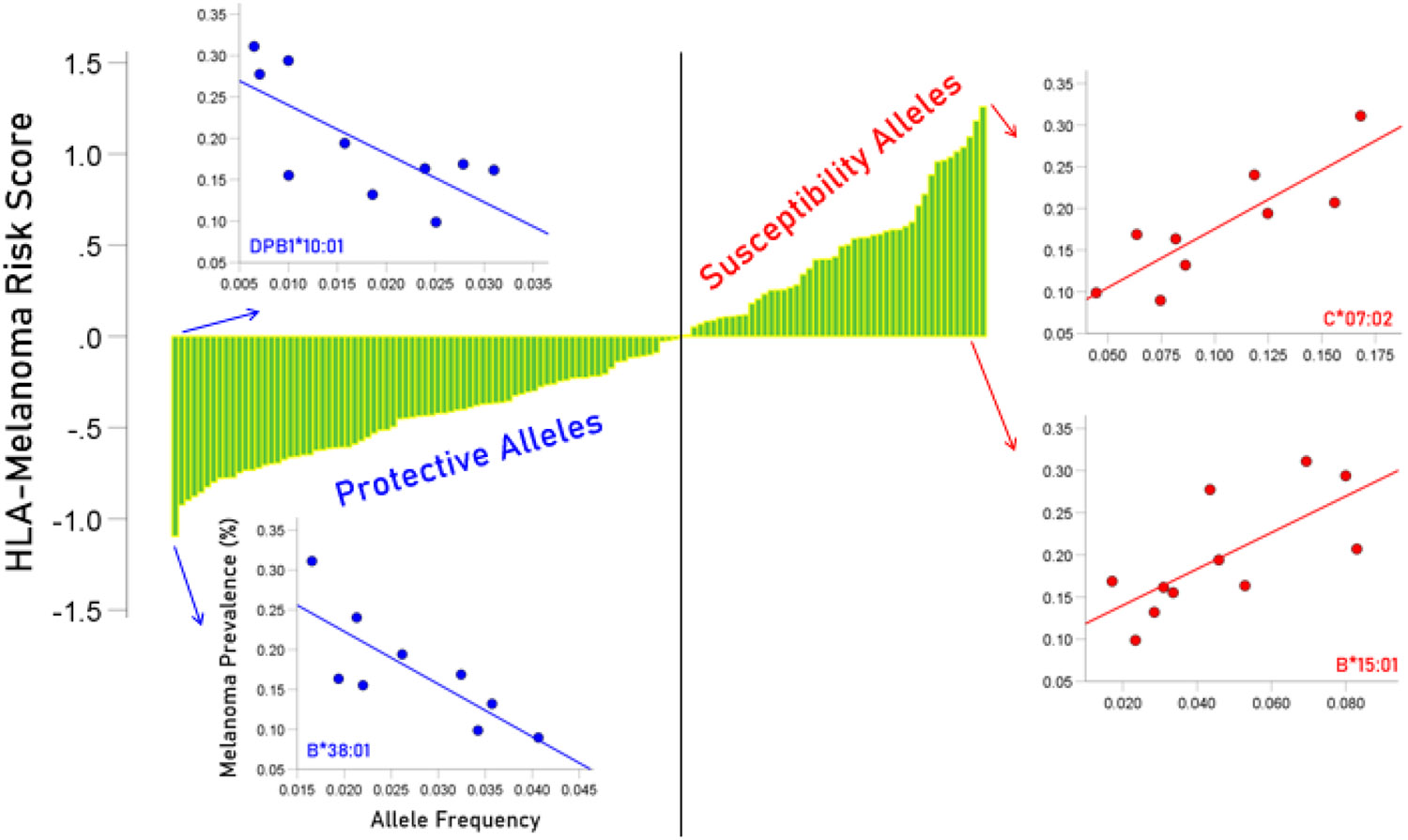
The HLA-melanoma risk scores are plotted against their rank. Arrows indicate the position in the graph of the plotted allele. Blue scatter plots illustrate the relation between the frequency of two protective alleles and melanoma frequency in CWE countries; for allele B*38:01, r = −0.799, $r^{\prime }$, *P* = 0.0098; for allele DPB1*10:01, r = −0.728, $r^{\prime }$, *P* = 0.017. Red scatter plots illustrate the relation between the frequency of two susceptibility alleles and melanoma frequency in CWE countries; for allele C*07:02, r = 0.851, $r^{\prime }$ = 1.259, *P* = 0.0036; for allele B*15:01, r = 0.777, $r^{\prime }$, *P* = 0.0082.

All IMS values are plotted against their rank in Fig. 1 together with scatter plots of two protective and two susceptibility alleles to illustrate the dependence of melanoma prevalence on allele frequency (negative for protective and positive for susceptibility alleles, color-coded in blue and red, respectively). Of the 127 alleles investigated, the frequencies of 79 alleles were negatively associated with melanoma prevalence, indicating a protective effect, whereas the frequencies of 48 alleles were positively associated with melanoma prevalence, indicating a susceptibility effect. The IMS scores of the protective alleles are given in Table 4 and those of the susceptibility alleles are given in Table 5. It can be seen that both types of alleles can be found in both HLA classes and all 6 classical genes.

The results of the statistical analysis of the proportions are given in Table 6. We found the following. (a) The overall proportion of protective alleles (79/127 = 0.622) was statistically significantly higher than the null hypothesis of the proportion = 0.5, P = 0.005, Wald test). (b) Of the 69 class I alleles, 47 were protective (proportion = 0.681, P = 0.001). (c) Within class I, alleles of gene B had an overall statistically significant protective effect (proportion = 25/36 = 0.694, P = 0.011). With respect to class II, there were no statistically significant overall effects (Table 6).

### Melanoma immunogenetic scores: Supertypes

The results of the statistical analysis of the proportions for 5 supertypes with N alleles > 5 (A01, A03, B07, B27, B44; Table 2) are given in Table 7. It can be seen that of the 5 supertypes tested, all but A03 comprised more protective than susceptibility alleles, although only in A01 and B27 this protective preponderance reached statistical significance.

### Melanoma immunogenetic score $r^{\prime }$: Application to individuals

The IMS scores analyzed above (Tables 4 and 5) refer to particular alleles. Given that an individual carries a total of 12 alleles (2 of each 6 HLA genes), the overall protection/susceptibility to melanoma for a specific individual is given by the average of the 12 IMS scores, one for each one of the 12 HLA alleles carried by that individual:

Overall Melanoma P/S Risk Score

(2) $$=\sum {\text{IMS}}^{A(1)}+{\text{IMS}}^{A(2)}+{\text{IMS}}^{B(1)}+{\text{IMS}}^{B(2)}+{\text{IMS}}^{C(1)}+{\text{IMS}}^{C(2)}+{\text{IMS}}^{\text{DPB1}(1)}+\;{\text{IMS}}^{\text{DPB}1(2)}+{\text{IMS}}^{\text{DQB}1(1)}+{\text{IMS}}^{\text{DQB}1(2)}+{\text{IMS}}^{\text{DRB}1(1)}+{\text{IMS}}^{\text{DRB}1(2)}$$

where the subscripts on genes denote the 2 pairs of alleles carried by an individual for each classical HLA gene.

### Melanoma immunogenetic score $R^{\prime }$: Application to populations and CBI assessment

A second IMS estimate is the expected HLA-melanoma P/S $R^{\prime }$ score, which is relevant to a population, where a given allele can be present in individuals together with 11 other alleles. More specifically, $R^{\prime }$ is an estimate of the P/S influence of a specific allele in the presence of random combinations of any additional 11 alleles. The estimates of the expected (long-term) values of $R^{\prime }$ are given in Table 8. It can be seen that most estimates are negative (i.e., protective), due to the fact that the IMS of most alleles are negative. This measure is especially relevant when evaluating effects of a given HLA allele on CBI outcomes because the individuals carrying the allele also carry 11 additional alleles which could also influence the outcome. The incorporation of the combined effect of 1000 random selections of these 11 alleles (from the total allele pool) in the derivation of $R^{\prime }$ (see Methods) makes this estimate a solid and realistic measure by which to gauge the effect of the allele on CBI outcome.

### Relation to findings from CBI cancer immunotherapy

Chowell et al.^8^ reported on an association between HLA supertypes and degree of success of CBI in melanoma (see Table 1 in ref.^8^). Of 12 supertypes tested, statistically significant favorable effects with respect to CBI outcomes were found for 2 supertypes (B62 and B44). In our study, supertype B62 comprised only 2 alleles (Table 2) and, hence, could not be tested, whereas B44 (with 11 alleles) showed a preponderance of protective alleles but did not reach statistical significance (Table 7). However, a more clear picture is obtained with regard to individual alleles. Specifically, it was reported^8^ that alleles B*18:01, B*44:02, B*44:03, B*44:05 and B*50:01 were associated with favorable CBI response, whereas B*15:01 was associated with poor response. It can be seen in Table 7 that there was a complete congruence between this effect on CBI outcome and the P/S property of $R^{\prime }$ of these alleles, namely that (a) 5 alleles with beneficial outcome had protective (negative) $R^{\prime }$ scores (in blue), and (b) one allele with poor outcome had susceptibility (positive) $R^{\prime }$score (in red).

## Discussion

HLA is instrumental in immunosurveillance and T cell activation aimed at protection against foreign antigens. Neoantigens, a product of genetic mutations resulting from carcinogenesis or viral infections, stimulate the immune system to attack cancer cells^4,5^. Indeed, tumor specific neoantigens, which are selectively expressed on tumor cells and are therefore considered non-self by the immune system and are unaffected by immune tolerance, have become an increasingly promising target for personalized cancer immunotherapy^29,30^. However, a prerequisite of neoantigen presentation to the cancer cell’s surface and subsequent stimulation of immune system activation is that the neoantigen possesses a binding motif that is recognized by an individual’s HLA^31^. With regard to CBI, for instance, HLA-related differences in treatment effectiveness ae attributed to varying ability to bind melanoma tumor neoantigens^8^. Presumably, B*18:01, B*44:02, B*44:03, B*44:05 and B*50:01, which were shown to have a beneficial effect on survival under CBI therapy bind with greater efficiency than alleles that were found to have a negative effect on survival such as B*15:01. Remarkably, there was a complete congruence between the effects of those alleles on survival outcome and their expected IMS value (color-coded alleles Table 7). Specifically, allele B*15:01, which had a negative effect on survival^8^ had a susceptibility $R^{\prime }$ score (red), whereas all alleles with positive effects on survival (B*18:01, B*44:02, B*44:03, B*44:05, B*50:01) had a protective IMS score (blue). These results suggest that HLA alleles that influence melanoma treatment (positively or negatively) also broadly influence melanoma protection or risk.

Finally, a word of caution regarding the interpretation of effects of HLA supertypes. Although the assignment of an allele to a supertype is based on sound biophysical principles^18^, this does not ensure a homogeneity of biological effect by the various alleles of a given supertype, given that even a single amino acid difference in a HLA molecule can result in a major difference in biological action^12^. In fact, the diversity of action of melanoma-related HLA molecules of the same supertype becomes evident from an examination of the confidence intervals of the hazard ratios (HR) of the effects of 12 HLA supertypes on melanoma CBI outcome^8^. Of these 12 supertypes, 10 did not show a statistically significant effect but had a wide range of 95% CI indicating the presence of mixed effects, i.e. of alleles with beneficial, neutral or detrimental effect on CBI outcome. For example, the lower and upper 95% CI of HR for supertype A01A03 (Table 1 in ref.^8^) were 0.43 and 2.94, respectively, with a fairly wide Confidence Limit Ratio^32^ $\left(CLR=\frac{\text{Upper}\;95\%\;\text{CI}}{\text{Lower}\;95\%\;\text{CI}}=6.84\right)$, indicating the inclusion in A01A03 of alleles with very different effects on CBI outcome. In all 10 supertypes above, HR 95% CI straddled the critical value of 1, thus indicating that all of these supertypes contained alleles with opposite effects on CBI outcome (beneficial/poor). Strictly speaking, the nonsignificant P values for the HR of these supertypes mean that the null hypothesis that HR = 1 cannot be rejected at α = 0.05, but the range in the 95% CI indicates the presence of mixed effects. The same considerations apply to our findings with respect to protective/susceptibility alleles and supertypes (Table 7), namely that supertypes comprise alleles with diverse effects. This brings to focus the point that the important unit for measuring HLA-related effects, in practically any application, is the individual allele, and not an aggregate of alleles, and, more specifically, the allele determined at 4-digit (high) resolution which distinguishes alleles encoding amino acid differences. This has been shown to be of critical importance in a recent large study of HLA associations in Myalgic Encephalomyelitis/Chronic Fatigue Syndrome (ME/CFS)^33^.

**Figure 2:**
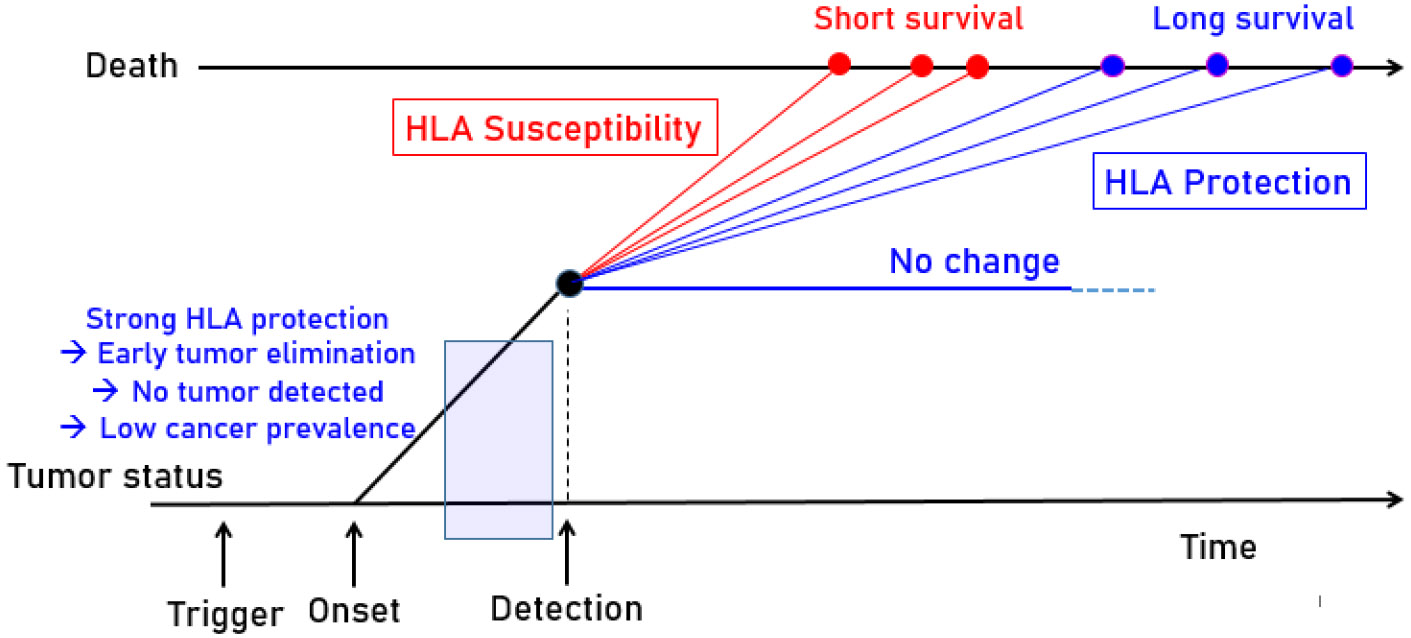
Schematic diagram to illustrate the hypothesized dependence of melanoma prevalence and survival on protective (blue) and susceptibility (red) HLA molecules. HLA alleles that are protective at the population level may facilitate melanoma binding and immunogenicity, potentially eliminating cancer cells even prior to detection; therefore, those protective alleles would be associated with low prevalence of melanoma. In contrast, HLA alleles that are associated with susceptibility may be unable to sufficiently bind and eliminate melanoma neoantigens, thereby promoting continued proliferation of cancerous cells and reduced survival.

In this study, we found other class I and class II alleles to have even more robust protective and susceptibility effects than those previously shown to influence CBI effectiveness^8^. Future studies are warranted to evaluate the extent to which the alleles that contribute to population protection or susceptibility translate to the individual level. Nonetheless, the current findings add to the literature documenting the importance not only of class I, but also class II alleles, on melanoma survival^3,34,35^ and extend those effects to the population. We hypothesize that a similar mechanism, namely antigen elimination, underlies the influence of HLA on melanoma at the individual and population level, as illustrated schematically in Fig. 2. Cancer cells notoriously evade the immune system via loss or alteration of HLA^36^. It is possible that some HLA alleles may be more vulnerable to cancer immune escape mechanisms, resulting in increased susceptibility, although that remains to be investigated.

The present study documents the influence of a large number of high-resolution HLA alleles on the population prevalence of melanoma in Continental Western Europe. While compelling, the findings must be considered in the context of several qualifications. First, the present findings come from data in 14 CWE countries and may not extend to populations in other earth locations. Second, HLA is the most highly polymorphic region of the human genome; thus, despite evaluating the influence of 127 HLA alleles on melanoma prevalence, many additional alleles not captured here may influence melanoma prevalence and immunotherapy outcome. Third, the present study exclusively focused on the influence of HLA on melanoma. Other factors including additional genetic contributors, host microbiome, and environmental factors have been shown to affect the appearance of melanoma and cancer immunity in general^31^and were not investigated here. Future studies evaluating potential moderating effects of HLA on factors that have been linked to cancer immunity are warranted but beyond the scope of the present study. Finally, the HLA-melanoma associations identified here are likely specific to melanoma. Analyses are underway to evaluate HLA associations with the prevalence of other cancers.

## Figures and Tables

**Table 1:** Distribution of 127 HLA alleles analyzed to Class and Gene.

	Class I (N = 69 alleles)			Class II (N = 58 alleles)		
Gene	A	B	C	DPB1	DQB1	DRB1
Count	20	36	13	15	14	29

**Table 2:** Distribution of 56 Class I A and B alleles in supertypes.

Supertype	Count
A01	6
A02	3
A03	6
A24	3
A103	1
A124	1
B07	8
B08	1
B27	8
B44	11
B58	3
B62	2
Unassigned	3
Total	56

**Table 3:** The 127 HLA alleles used and their Class, gene and supertype assignments.

Index	Allele	Class	Gene	Supertype
1	A*01:01	I	A	A01
2	A*02:01	I	A	A02
3	A*02:05	I	A	A02
4	A*03:01	I	A	A03
5	A*11:01	I	A	A03
6	A*23:01	I	A	A24
7	A*24:02	I	A	A24
8	A*25:01	I	A	A01
9	A*26:01	I	A	A01
10	A*29:01	I	A	A24
11	A*29:02	I	A	A01 A24
12	A*30:01	I	A	A01 A03
13	A*30:02	I	A	A01
14	A*31:01	I	A	A03
15	A*32:01	I	A	A01
16	A*33:01	I	A	A03
17	A*33:03	I	A	A03
18	A*36:01	I	A	A01
19	A*68:01	I	A	A03
20	A*68:02	I	A	A02
21	B*07:02	I	B	B07
22	B*08:01	I	B	B08
23	B*13:02	I	B	Unassigned
24	B*14:01	I	B	B27
25	B*14:02	I	B	B27
26	B*15:01	I	B	B62
27	B*15:17	I	B	B58
28	B*15:18	I	B	B27
29	B*18:01	I	B	B44
30	B*27:02	I	B	B27
31	B*27:05	I	B	B27
32	B*35:01	I	B	B07
33	B*35:02	I	B	B07
34	B*35:03	I	B	B07
35	B*35:08	I	B	B07
36	B*37:01	I	B	B44
37	B*38:01	I	B	B27
38	B*39:01	I	B	B27
39	B*39:06	I	B	B27
40	B*40:01	I	B	B44
41	B*40:02	I	B	B44
42	B*41:01	I	B	B44
43	B*41:02	I	B	B44
44	B*44:02	I	B	B44
45	B*44:03	I	B	B44
46	B*44:05	I	B	B44
47	B*45:01	I	B	B44
48	B*47:01	I	B	Unassigned
49	B*49:01	I	B	Unassigned
50	B*50:01	I	B	B44
51	B*51:01	I	B	B07
52	B*52:01	I	B	B62
53	B*55:01	I	B	B07
54	B*56:01	I	B	B07
55	B*57:01	I	B	B58
56	B*58:01	I	B	B58
57	C*01:02	I	C	
58	C*03:03	I	C	
59	C*04:01	I	C	
60	C*05:01	I	C	
61	C*06:02	I	C	
62	C*07:01	I	C	
63	C*07:02	I	C	
64	C*07:04	I	C	
65	C*12:02	I	C	
66	C*12:03	I	C	
67	C*14:02	I	C	
68	C*15:02	I	C	
69	C*16:01	I	C	
70	DPB1*01:01	II	DPB1	
71	DPB1*02:01	II	DPB1	
72	DPB1*02:02	II	DPB1	
73	DPB1*03:01	II	DPB1	
74	DPB1*04:01	II	DPB1	
75	DPB1*04:02	II	DPB1	
76	DPB1*05:01	II	DPB1	
77	DPB1*06:01	II	DPB1	
78	DPB1*09:01	II	DPB1	
79	DPB1*10:01	II	DPB1	
80	DPB1*11:01	II	DPB1	
81	DPB1*13:01	II	DPB1	
82	DPB1*14:01	II	DPB1	
83	DPB1*17:01	II	DPB1	
84	DPB1*19:01	II	DPB1	
85	DQB1*02:01	II	DQB1	
86	DQB1*02:02	II	DQB1	
87	DQB1*03:01	II	DQB1	
88	DQB1*03:02	II	DQB1	
89	DQB1*03:03	II	DQB1	
90	DQB1*04:02	II	DQB1	
91	DQB1*05:01	II	DQB1	
92	DQB1*05:02	II	DQB1	
93	DQB1*05:03	II	DQB1	
94	DQB1*06:01	II	DQB1	
95	DQB1*06:02	II	DQB1	
96	DQB1*06:03	II	DQB1	
97	DQB1*06:04	II	DQB1	
98	DQB1*06:09	II	DQB1	
99	DRB1*01:01	II	DRB1	
100	DRB1*01:02	II	DRB1	
101	DRB1*01:03	II	DRB1	
102	DRB1*03:01	II	DRB1	
103	DRB1*04:01	II	DRB1	
104	DRB1*04:02	II	DRB1	
105	DRB1*04:03	II	DRB1	
106	DRB1*04:04	II	DRB1	
107	DRB1*04:05	II	DRB1	
108	DRB1*04:07	II	DRB1	
109	DRB1*04:08	II	DRB1	
110	DRB1*07:01	II	DRB1	
111	DRB1*08:01	II	DRB1	
112	DRB1*08:03	II	DRB1	
113	DRB1*09:01	II	DRB1	
114	DRB1*10:01	II	DRB1	
115	DRB1*11:01	II	DRB1	
116	DRB1*11:02	II	DRB1	
117	DRB1*11:03	II	DRB1	
118	DRB1*11:04	II	DRB1	
119	DRB1*12:01	II	DRB1	
120	DRB1*13:01	II	DRB1	
121	DRB1*13:02	II	DRB1	
122	DRB1*13:03	II	DRB1	
123	DRB1*13:05	II	DRB1	
124	DRB1*14:01	II	DRB1	
125	DRB1*15:01	II	DRB1	
126	DRB1*15:02	II	DRB1	
127	DRB1*16:01	II	DRB1	

**Table 4:** Melanoma immunogenetic scores for the 79 HLA protective alleles ranked from high to low Protection.

Index	Allele	Class	Gene	IMS $\left(r^{\prime }\right)$
1	B*38:01	I	B	−1.095
2	DPB1*10:01	II	DPB1	−0.924
3	B*49:01	I	B	−0.898
4	DRB1*01:02	II	DRB1	−0.876
5	B*18:01	I	B	−0.855
6	DPB1*02:01	II	DPB1	−0.826
7	B*51:01	I	B	−0.799
8	DPB1*13:01	II	DPB1	−0.777
9	C*04:01	I	C	−0.775
10	B*35:08	I	B	−0.773
11	DPB1*14:01	II	DPB1	−0.747
12	B*41:02	I	B	−0.734
13	C*12:03	I	C	−0.732
14	A*23:01	I	A	−0.722
15	DRB1*11:03	II	DRB1	−0.705
16	B*35:03	I	B	−0.698
17	DRB1*04:03	II	DRB1	−0.694
18	A*32:01	I	A	−0.674
19	C*14:02	I	C	−0.662
20	DRB1*13:05	II	DRB1	−0.659
21	B*50:01	I	B	−0.649
22	DQB1*05:02	II		−0.648
23	DQB1*03:01	II	DQB1	−0.625
24	DRB1*04:02	II	DRB1	−0.622
25	A*11:01	I	A	−0.614
26	DRB1*13:03	II	DRB1	−0.610
27	DRB1*15:02	II	DRB1	−0.608
28	A*33:03	I	A	−0.607
29	A*26:01	I	A	−0.590
30	B*35:02	I	B	−0.572
31	C*15:02	I	C	−0.554
32	DRB1*11:02	II	DRB1	−0.530
33	C*12:02	I	C	−0.514
34	B*44:05	I	B	−0.512
35	B*15:18	I	B	−0.495
36	DRB1*07:01	II	DRB1	−0.452
37	B*14:02	I	B	−0.448
38	DRB1*11:04	II	DRB1	−0.444
39	DRB1*16:01	II	DRB1	−0.437
40	A*36:01	I	A	−0.435
41	B*58:01	I	B	−0.432
42	A*33:01	I	A	−0.422
43	A*02:05	I	A	−0.421
44	DQB1*05:03	II	DQB1	−0.413
45	B*14:01	I	B	−0.404
46	DPB1*02:02	II	DPB1	−0.400
47	B*52:01	I	B	−0.389
48	A*30:02	I	A	−0.379
49	A*29:02	I	A	−0.371
50	DRB1*11:01	II	DRB1	−0.369
51	B*44:03	I	B	−0.365
52	B*41:01	I	B	−0.362
53	DRB1*01:03	II	DRB1	−0.354
54	DQB1*06:01	II	DQB1	−0.325
55	B*47:01	I	B	−0.316
56	DQB1*02:02	II	DQB1	−0.305
57	B*45:01	I	B	−0.299
58	DPB1*17:01	II	DPB1	−0.275
59	DRB1*14:01	II	DRB1	−0.265
60	DRB1*04:05	II	DRB1	−0.261
61	C*07:04	I	C	−0.245
62	A*01:01	I	A	−0.240
63	DRB1*04:07	II	DRB1	−0.228
64	DPB1*09:01	II	DPB1	−0.227
65	DQB1*06:09	II	DQB1	−0.226
66	B*15:17	I	B	−0.218
67	B*39:06	I	B	−0.217
68	DRB1*08:03	II	DRB1	−0.206
69	B*39:01	I	B	−0.174
70	B*57:01	I	B	−0.139
71	DPB1*06:01	II	DPB1	−0.135
72	C*16:01	I	C	−0.116
73	A*68:02	I	A	−0.112
74	B*27:02	I	B	−0.108
75	B*35:01	I	B	−0.101
76	A*30:01	I	A	−0.088
77	DRB1*03:01	II	DRB1	−0.026
78	C*06:02	I	C	−0.022
79	A*29:01	I	A	−0.015

**Table 5:** Melanoma immunogenetic scores for the 48 HLA susceptibility alleles ranked from high to low susceptibility.

Index	Allele	Class	Gene	$\mathrm{IMS}(r^{\prime })$
1	C*07:02	I	C	1.259
2	B*37:01	I	B	1.181
3	DRB1*04:01	II	DRB1	1.093
4	B*15:01	I	B	1.037
5	B*07:02	I	B	1.013
6	A*31:01	I	A	0.982
7	DRB1*15:01	II	DRB1	0.965
8	B*40:01	I	B	0.957
9	DPB1*01:01	II	DPB1	0.885
10	A*03:01	I	A	0.779
11	DQB1*03:02	II	DQB1	0.717
12	DPB1*04:01	II	DPB1	0.627
13	DRB1*04:04	II	DRB1	0.603
14	DQB1*03:03	II	DQB1	0.585
15	DQB1*06:02	II	DQB1	0.584
16	DRB1*01:01	II	DRB1	0.576
17	DRB1*04:08	II	DQB1	0.560
18	DQB1*02:01	II	DQB1	0.555
19	DQB1*06:04	II	DQB1	0.543
20	C*03:03	I	C	0.542
21	DRB1*13:02	II	DRB1	0.536
22	B*27:05	I	B	0.502
23	B*55:01	I	B	0.494
24	DQB1*04:02	II	DQB1	0.437
25	DRB1*08:01	II	DRB1	0.424
26	DRB1*09:01	II	DRB1	0.423
27	B*08:01	I	B	0.420
28	DRB1*12:01	II	DRB1	0.372
29	DRB1*13:01	II	DRB1	0.340
30	A*24:02	I	A	0.282
31	DPB1*05:01	II	DPB1	0.268
32	B*40:02	I	B	0.256
33	DQB1*06:03	II	DQB1	0.254
34	A*02:01	I	A	0.252
35	B*56:01	I	B	0.232
36	DPB1*19:01	II	DPB1	0.204
37	C*05:01	I	C	0.181
38	C*07:01	I	C	0.118
39	B*13:02	I	B	0.115
40	DPB1*03:01	II	DPB1	0.109
41	B*44:02	I	B	0.108
42	C*01:02	I	C	0.102
43	A*68:01	I	A	0.087
44	A*25:01	I	A	0.082
45	DRB1*10:01	II	DRB1	0.068
46	DQB1*05:01	II	DQB1	0.052
47	DPB1*11:01	II	DPB1	0.008
48	DPB1*04:02	II	DPB1	0.003

**Table 6:** Number of melanoma protective and susceptibility alleles in HLA Class I, II and their classical genes. Confidence intervals are Agresti-Coull. Statistically significant results are marked by*.

	Gene	Total N	N protective	N susceptibility	Proportion protective	Lower 95% CI	Upper 95% CI	Z	Wald Test (2 sided P)
Class I	A	20	14	6	0.700	0.479	0.857	1.952	0.051
	B	36	25	11	0.694	0.530	0.821	2.533	0.011*
	C	13	8	5	0.615	0.354	0.824	0.855	0.392
	Total	69	47	22	0.681	0.564	0.779	3.229	0.001*
Class II	DPB1	15	8	7	0.533	0.301	0.752	0.259	0.796
	DQB1	14	6	8	0.429	0.213	0.674	0.540	0.589
	DRB1	29	18	11	0.621	0.439	0.774	1.339	0.180
	Total	58	32	26	0.552	0.424	0.673	0.792	0.428
Total		127	79	48	0.622	0.535	0.702	2.837	0.005*

**Table 7:** Number of melanoma protective and susceptibility alleles in 5 HLA Class I supertypes with N > 5. Confidence intervals are Agresti-Couli. Statistically significant results are colored red and marked by*.

Supertype	N	N protective	N susceptibility	Proportion protective	Lower 95% CI	Upper 95% CI	Z	Wald Test (2 sided P)
A01	6	5	1	0.833	0.448	0.989	2.191	0.028*
A03	6	3	3	0.500	0.188	0.912	0.000	1.000
B07	8	5	3	0.625	0.304	0.865	0.730	0.465
B27	8	7	1	0.875	0.508	0.999	3.207	0.001*
B44	11	7	7	0.636	0.362	0.950	0.940	0.347

**Table 8:** Expected P/S estimates $R^{\prime }$for the 127 alleles investigated in alphabetical order. The 6 alleles for which an effect on melanoma CBI immunotherapy outcome has been reported ^8^ are in bold and colored red to indicate poor outcome and blue to indicate beneficial outcome. Notice that these CBI-based attributes correspond to susceptibility and protective $R^{\prime }$, respectively.

	Allele	Expected P/S estimate $R^{\prime }$
1	A*01:01	−0.09810
2	A*02:01	−0.06601
3	A*02:05	−0.11706
4	A*03:01	−0.02287
5	A*11:01	−0.13304
6	A*23:01	−0.14128
7	A*24:02	−0.06191
8	A*25:01	−0.06935
9	A*26:01	−0.13616
10	A*29:01	−0.08055
11	A*29:02	−0.10928
12	A*30:01	−0.08787
13	A*30:02	−0.11728
14	A*31:01	0.00081
15	A*32:01	−0.12997
16	A*33:01	−0.11542
17	A*33:03	−0.12972
18	A*36:01	−0.11615
19	A*68:01	−0.06584
20	A*68:02	−0.08632
21	B*07:02	0.00323
22	B*08:01	−0.05063
23	B*13:02	−0.07745
24	B*14:01	−0.11786
25	B*14:02	−0.11589
26	B*15:01	0.00777
27	B*15:17	−0.10458
28	B*15:18	−0.12400
29	B*18:01	−0.15214
30	B*27:02	−0.09829
31	B*27:05	−0.04446
32	B*35:01	−0.09081
33	B*35:02	−0.13615
34	B*35:03	−0.14199
35	B*35:08	−0.15024
36	B*37:01	0.01478
37	B*38:01	−0.17444
38	B*39:01	−0.09179
39	B*39:06	−0.10054
40	B*40:01	−0.00427
41	B*40:02	−0.06165
42	B*41:01	−0.11417
43	B*41:02	−0.1469
44	B*44:02	−0.07697
45	B*44:03	−0.11213
46	B*44:05	−0.12451
47	B*45:01	−0.10965
48	B*47:01	−0.10636
49	B*49:01	−0.15750
50	B*50:01	−0.13779
51	B*51:01	−0.14575
52	B*52:01	−0.12171
53	B*55:01	−0.03946
54	B*56:01	−0.06161
55	B*57:01	−0.09192
56	B*58:01	−0.12279
57	C*01:02	−0.08364
58	C*03:03	−0.04563
59	C*04:01	−0.15210
60	C*05:01	−0.07476
61	C*06:02	−0.08138
62	C*07:01	−0.07739
63	C*07:02	0.01269
64	C*07:04	−0.12142
65	C*12:02	−0.12683
66	C*12:03	−0.15016
67	C*14:02	−0.14688
68	C*15:02	−0.13256
69	C*16:01	−0.09218
70	DPB1*01:01	−0.00218
71	DPB1*02:01	−0.14851
72	DPB1*02:02	−0.11065
73	DPB1*03:01	−0.07555
74	DPB1*04:01	−0.03509
75	DPB1*04:02	−0.08794
76	DPB1*05:01	−0.06835
77	DPB1*06:01	−0.09915
78	DPB1*09:01	−0.10201
79	DPB1*10:01	−0.15335
80	DPB1*11:01	−0.08137
81	DPB1*13:01	−0.14686
82	DPB1*14:01	−0.14786
83	DPB1*17:01	−0.11460
84	DPB1*19:01	−0.06244
85	DQB1*02:01	−0.05403
86	DQB1*02:02	−0.12581
87	DQB1*03:01	−0.16130
88	DQB1*03:02	−0.04474
89	DQB1*03:03	−0.04820
90	DQB1*04:02	−0.06698
91	DQB1*05:01	−0.09846
92	DQB1*05:02	−0.15057
93	DQB1*05:03	−0.13746
94	DQB1*06:01	−0.13615
95	DQB1*06:02	−0.05035
96	DQB1*06:03	−0.08316
97	DQB1*06:04	−0.05670
98	DQB1*06:09	−0.12162
99	DRB1*01:01	−0.04449
100	DRB1*01:02	−0.17229
101	DRB1*01:03	−0.12101
102	DRB1*03:01	−0.09058
103	DRB1*04:01	0.00772
104	DRB1*04:02	−0.14398
105	DRB1*04:03	−0.15780
106	DRB1*04:04	−0.04321
107	DRB1*04:05	−0.10505
108	DRB1*04:07	−0.10413
109	DRB1*04:08	−0.03819
110	DRB1*07:01	−0.13275
111	DRB1*08:01	−0.06066
112	DRB1*08:03	−0.11381
113	DRB1*09:01	−0.05353
114	DRB1*10:01	−0.09050
115	DRB1*11:01	−0.12326
116	DRB1*11:02	−0.13365
117	DRB1*11:03	−0.14712
118	DRB1*11:04	−0.13190
119	DRB1*12:01	−0.05823
120	DRB1*13:01	−0.06099
121	DRB1*13:02	−0.04509
122	DRB1*13:03	−0.14444
123	DRB1*13:05	−0.14817
124	DRB1*14:01	−0.11612
125	DRB1*15:01	0.00101
126	DRB1*15:02	−0.13911
127	DRB1*16:01	−0.12667
